# Vector Competence of *Culicoides sonorensis* (Diptera: Ceratopogonidae) for Epizootic Hemorrhagic Disease Virus Serotype 2 Strains from Canada and Florida

**DOI:** 10.3390/v11040367

**Published:** 2019-04-22

**Authors:** Bethany L. McGregor, Dinesh Erram, Carolina Acevedo, Barry W. Alto, Nathan D. Burkett-Cadena

**Affiliations:** Florida Medical Entomology Laboratory, University of Florida, 200 9th St. SE, Vero Beach, FL 32962, USA; derram@ufl.edu (D.E.); c.acevedo@ufl.edu (C.A.); bwalto@ufl.edu (B.W.A.); nburkettcadena@ufl.edu (N.D.B.-C.)

**Keywords:** *Culicoides sonorensis*, epizootic hemorrhagic disease virus, EHDV-2, vector competence, strain, infection, dissemination, transmission

## Abstract

Epizootic hemorrhagic disease virus (EHDV), an *Orbivirus* transmitted by *Culicoides* spp. vectors, is represented by seven serotypes and numerous strains worldwide. While studies comparing vector competence between serotypes exist, studies between viral strains are lacking. In this study, we examined the rates of infection, dissemination, and transmission of two strains of EHDV-2 orally fed to the known vector, *Culicoides sonorensis* Wirth & Jones. *Culicoides sonorensis* cohorts were fed an infectious blood meal containing EHDV-2 strains from either Alberta, Canada (Can-Alberta) or Florida (5.5 log_10_ PFUe/mL) and tested for the vector’s susceptibility to infection and dissemination. In addition, transmission rates of the virus were assessed and compared using capillary tube and honey card methods. Our results show that the Florida strain had higher infection and dissemination rates than the Can-Alberta strain in spite of the Florida strain having significantly lower viral titers in *C. sonorensis* bodies, legs, and saliva than the Can-Alberta strain. Overall transmission rates were not significantly different between the two strains but varied significantly between the methods used. These findings suggest that the consequences of EHDV infection in *C. sonorensis* vary between virus strains and have huge implications in future vector competence studies involving *Culicoides* species and Orbiviruses.

## 1. Introduction

Epizootic hemorrhagic disease virus (EHDV) (Genus *Orbivirus*, Family Reoviridae) is a *Culicoides*-borne virus that affects a variety of domestic and wild ungulates of the families Cervidae and Bovidae. Infection of susceptible hosts with this pathogen can result in economically significant negative effects including decreased milk production, mouth lesions, and mortality in cattle [1,2], antler growth abnormalities and mortality in mule deer [3,4], and considerable mortality, chronic lesions and hoof abnormalities in white-tailed deer [2,5]. Despite the huge impact of EHDV in animal agriculture, many fundamental aspects of EHDV transmission are still understudied.

Currently, three EHDV serotypes are known to occur in the USA: EHDV-1, EHDV-2, and EHDV-6 [6,7,8], with serotype determination primarily based on two outer capsid proteins, VP2 and VP5 [9]. However, within these serogroups exist numerous geographically distinct viral strains that are characterized by a vast amount of genetic heterogeneity, which contributes to high rates of evolution amongst these strains. Interestingly, documentation from other pathogen systems shows that even minor genetic variation between virus strains can have significant effects on infection and transmission dynamics in arthropod vectors and hosts [10,11,12]. For example, the emergence of Chikungunya virus (CHIKV) in the Old World was attributed to a single amino acid change in the viral E1 envelope glycoprotein gene, which allowed *Aedes albopictus* Skuse (previously considered an inefficient vector) to become a primary vector of CHIKV in the Indian Ocean epidemic during 2004–2007. This mutation reduced the extrinsic incubation period (the period from acquisition of virus until transmission is possible) of CHIKV and increased the vector competence of *Ae. albopictus* by 1000-fold [12,13]. A similar situation exists among enzootic Venezuelan equine encephalitis viruses (VEEV) that usually are not capable of equine amplification because they do not produce adequate viremia. However, amino acid replacements in the E2 envelope glycoprotein have been linked to enhanced virus amplification in equines that most likely resulted in the emergence of epidemic VEEV [14,15,16]. Significant variation between virus strains has also been found in the *Culicoides*-borne *Orbivirus* African horse sickness virus (AHSV), where the vector, *Culicoides imicola* Kieffer, exhibited significant variation in infection rates for multiple strains of AHSV serotypes 1, 3, and 7 [17]. Taken together, these findings suggest that geographic strains of arboviruses differ in their ability to replicate and negotiate barriers to infection/transmission in arthropod vectors and demonstrate the need for additional evaluations of strain-level variation among arboviruses.

While studies on the effect of viral strain in arthropod vectors for EHDV are lacking, there is evidence that different strains of EHDV can have novel characteristics and produce variable outcomes for the infected vertebrate hosts. One example of this phenomenon is the Ibaraki disease, caused by a series of EHDV-2 strains isolated from Japan [18]. Ibaraki disease causes greater than normal morbidity and mortality in cattle, a host that is generally not affected by other EHDV-2 strains [19]. An outbreak of Ibaraki resulted in numerous stillbirths and abortions in cattle [20], in stark contrast to the typical asymptomatic nature of EHDV-2 infection in cattle. Despite an extreme divergence of signs between typical EHDV-2 and Ibaraki in cattle, genetic differences between the strains were minor, resulting from a slight reassortment of viral RNA segment 2 [21].

Currently, only one confirmed vector of EHDV is known in the United States: *Culicoides sonorensis* Wirth and Jones [22,23], although other species likely transmit EHDV, particularly in the Southeastern United States, where *C. sonorensis* is rare or absent [24]. Throughout its range (Western USA, Northern Mexico, and Southern Canada), *C. sonorensis* populations are likely exposed to many different strains of each EHDV serotype annually. Despite this, most vector competency studies typically evaluate a single EHDV strain and interpret those results broadly, which has the potential to underestimate or overestimate competence.

The primary host of EHDV in North America is white-tailed deer (*Odocoileus virginianus*), a species which often demonstrates great morbidity and mortality associated with this pathogen [25]. At the same time, white-tailed deer farming is a growing industry in the United States with an estimated economic impact of $7.9 billion US dollars, supporting over 56,000 jobs [26]. As such, the impact of EHDV on white-tailed deer health and the white-tailed deer farming industry is tremendous, with EHDV associated mortality rates in white-tailed deer herds ranging from 60–80% [27,28]. Therefore, the resulting economic losses and overall negative impact on the cervid farming industry necessitate increased research into understanding the dynamics of EHDV transmission and various aspects of the competence of known EHDV vectors in the United States.

The aim of this study was two-fold. The first goal was to compare the susceptibility of *C. sonorensis* to infection, disseminated infection, and transmission potential of two strains of EHDV-2: A reference strain of the virus collected in Alberta, Canada, in 1962 (Can-Alberta strain), and a strain recently isolated from the spleen of a white-tailed deer that succumbed to the disease in Gadsden County, Florida, in 2016 (Florida strain). The second goal was to compare the efficacy of honey card and capillary tube bioassays to assess virus transmission potential of *C. sonorensis*. Honey cards were originally developed in Australia as a proxy method to monitor pathogen prevalence in trapped field collections of mosquitoes in the absence of sentinel animals [29]. This methodology has since been adapted in various arthropod vector-competence studies to estimate arbovirus transmission rates through collection and testing of saliva for the presence of viral RNA [30,31].

The main conclusions of this study indicate that viral strain has a significant effect on aspects of vector competence in *C. sonorensis*. When possible, efforts should be made to use strains representative of the geographic area under study. Furthermore, the use of a single transmission bioassay may result in false negatives and determinations of transmission potential are more accurate when multiple saliva collection methods are employed.

## 2. Materials and Methods

### 2.1. Culicoides Sonorensis Colony

Laboratory-reared *C. sonorensis*, from the Ausman colony maintained by the Arthropod-borne Animal Diseases Research Unit (ABADRU) of the United States Department of Agriculture (USDA, Agricultural Research Service) were shipped overnight to the Florida Medical Entomology Laboratory as 1-day old adults [32]. Midges were immediately aspirated into clean 500.0 mL paper cups with no-see-um netting (greatest mesh opening 0.6 mm) and moved to an incubator held at 25 ± 1 °C, 60–80% RH, and 12:12 light:dark (L:D) cycle until blood feeding the same day. No sugar was provided between shipping and blood feeding.

### 2.2. Viral Strains

Two EHDV-2 viral strains were used for this study: A reference strain isolated in Alberta, Canada, in 1962, obtained from the Centers for Disease Control and Prevention (CDC) (NCBI Accession #AM74997-AM745006), and a strain isolated from the spleen of a white-tailed deer that succumbed to the pathogen in Gadsden County, Florida, USA in 2016 (NCBI Accession #MF688816-MF688825). Both viruses were passaged twice on African green monkey kidney (Vero) cells maintained on medium 199 with Earle’s Balanced Salt Solution (HyClone Medium 199, GE Healthcare Life Sciences, Logan, UT, USA) containing 10% fetal bovine serum (Atlanta Biologicals, Flowery Branch, GA, USA), 2% penicillin streptomycin (Thermo Fisher Scientific, Waltham, MA, USA) and 0.2% Amphotericin B (Thermo Fisher Scientific).

### 2.3. Viral Infection

Cohorts of ≤50 midges were fed EHDV-2 infected blood using a Hemotek membrane feeding system (Hemotek Ltd., Blackburn, UK) following published protocols [33]. Parafilm was used as the membrane for blood feeding using defibrinated bovine blood (Hemostat laboratories, Dixon, CA, USA) in three treatments: Can-Alberta strain, Florida strain, and control (blood without the addition of virus). Blood was collected before and after each blood feeding trial to measure viral titer and to ensure titers were similar between the two infected groups. The calculated titer of virus in the two virus fed treatments were each 5.5 log_10_ plaque forming unit equivalents (PFUe)/mL. Midges were allowed to blood feed for one hour after which blood-fed females were sorted from the males and unfed females using a CO_2_ pad (LabScientific Carbon Dioxide Staging Platform, Cat #BGSU-12, Highlands, NJ, USA) and a stereoscopic light microscope (Nikon SMZ 745). Blood-engorged females were sorted into cups of ≤25 individuals, separated by treatment. After blood-feeding, midges were provided 10% sucrose solution *ad libitum* on cotton pads and maintained at 25 ± 1 °C, 60–80% RH, and 12:12 (L:D) cycle until processing.

### 2.4. Insect Tissue/Saliva Collection for Viral RNA Detection

Following the incubation period, *Culicoides* samples were dissected and bodies, legs, and saliva were tested for susceptibility to infection, disseminated infection, and transmission potential, respectively. Starting from day 3 post-infection, cohorts of midges from each viral treatment were selected at random days for sample collection. On average, daily cohorts consisted of approximately 15 Can-Alberta strain individuals and 21 Florida strain individuals. The greater cohort size for Florida strain infected midges reflects greater blood-feeding success with this treatment group. Legs and bodies of midges were dissected and placed into separate 1.5 mL microcentrifuge tubes containing 150.0 µL medium 199 and 5–10 2.0 mm borosilicate glass beads (Sigma-Aldrich, St. Louis, MO, USA) for homogenization.

#### 2.4.1. Capillary Tube Bioassays

Beginning from day 5, capillary tube assays were performed on the midges daily to collect their saliva. Upon removal of legs and wings, a 70.0 µL capillary tube containing a small amount of immersion oil was applied to the mouthparts of each midge to collect saliva [34,35]. To induce salivation, a 0.01% solution of malathion in acetone was applied to the thorax of each individual [36]. Midges were left to salivate into capillary tubes for one hour. Subsequently, immersion oil containing saliva was removed into 1.5 mL microcentrifuge tubes containing 150.0 µL of medium 199 [37].

#### 2.4.2. Honey Card Bioassays

Honey cards were used alongside capillary assays to compare the two methods for saliva collection/viral RNA detection in *C. sonorensis*. Thus, the same individual midges were tested by both methods. Starting on day 6 post-infection, midges in cups planned for collection the next day were anesthetized using CO_2_ and transferred individually to small cages modified from 50.0 mL conical tubes (Corning Brand, Corning, NY, USA). Modifications included cutting off the bottom of the tube at the 30.0 mL mark which was then covered with mesh and lining the interior with laboratory tape. Each midge was then given a small piece of filter paper (Whatman grade 1 filter paper, cut to <1 cm^2^) soaked in honey dyed blue with food coloring (McCormick, & Co. Inc., Hunt Valley, MD, USA) [30,38]. Prior to dissection the following day, midges were inspected visually for blue crop coloration to indicate ingestion of blue honey. Honey cards of midges that displayed blue coloration in the crop were collected into 1.5 mL microcentrifuge tubes containing 150.0 µL medium 199. For individuals that received both saliva collection assays, honey cards were administered the day prior to dissection and capillary assays.

### 2.5. Sample Processing and Viral RNA Detection

Body and leg samples of the midges collected were homogenized using the Bullet Blender Storm (Next Advance, Troy, NY, USA) for five minutes, following the manufacturer’s protocol. Honey cards were processed by crushing the filter paper with a pestle to release the virus into solution. Viral RNA was extracted using the Qiagen viral RNA mini kit (Cat#52906, Qiagen, Hilden, Germany) following manufacturer protocols. All body, leg, honey card, and capillary samples were processed separately. Unique ID numbers were assigned to each midge to monitor each assay (infection, dissemination, transmission) on an individual level.

All samples were screened on quantitative reverse-transcription polymerase chain reaction (qRT-PCR) using the SuperScript III One-step qRT-PCR kit (Thermo Fisher Scientific). Reaction reagents included 2.2 µL molecular grade water, 1.0 µL 10.0 µM forward primer, 1.0 µL 10.0 µM reverse primer, 10.0 µL 2× reaction mix, 0.4 µL EHDV probe, 0.4 µL Platinum *Taq*/SuperScript III reverse transcriptase mix, and 5.0 µL RNA template. Primers and probe sequences used were from Wernike et al. [39] (F: AAA AAG TTC YTC GTC GAC TGC, R: ATT GGC RTA RTA ACT GTT CAT GTT, Pr: ATC GAG ATG GAR CGC TTY TTG AGA AAA T). Reaction conditions were modified from Wilson et al. [40] to 25 min at 55 °C, 2 min at 95 °C, and 45 cycles of 10 s at 95 °C and 1 min at 57 °C. The limitations for detection of the two viral strains were determined at Cq = 35 for the Florida strain and Cq = 37 for the Can-Alberta strain. Limitations of detection were determined through the production of viral standards based on serially diluted virus samples. This approach allowed us to generate a standard curve to express the titer of EHDV in *Culicoides* samples by comparing cDNA synthesis for serial dilutions of EHDV in parallel with plaque assays to express viral titer as PFUe/mL (Supplementary File 1) [41].

### 2.6. Statistical Analysis

Contingency table analyses were run to test for strain, time, and strain by time interactions for bodies, legs, and saliva (honey cards and capillary tube assay positives combined). Legs were only tested if the body of the same individual already tested positive for viral RNA. As such, disseminated infection rates were calculated as number of positive leg samples out of the number of virus-positive bodies detected. Saliva was only tested if both legs and bodies tested positive for viral RNA and transmission rates were calculated by dividing total midges with detectable virus in saliva by total midges with disseminated infection.

Scatterplots were generated and linear regression analyses were run for each comparison to explore relationships between viral titers in bodies-legs and legs-saliva. Welch’s *t*-tests were employed to test for significant differences in viral titers between viral strains in bodies, legs, and saliva. For *t*-tests, data were analyzed in two ways: (1) Data aggregated by strain regardless of collection date and (2) data aggregated by strain and early (day 3-day 7) and late (day 8-day 13) periods. These periods were chosen based on an analysis of the progression of EHDV infections in *C. sonorensis* indicating that the number of virus particles reaches a maximum titer between 8–10 days post-feeding [42].

Chi-square test of independence was run on honey card and capillary assay positives between viral strains to determine whether results were independent of strain. Results were found to be dependent on strain, so separate Chi-square analyses were used for each viral strain to determine whether there were differences in the number of positives due to saliva collection method used. Viral titers were compared between methods for Florida and Can-Alberta strains using Welch’s two-sided *t*-tests.

## 3. Results

### 3.1. Infection, Dissemination, and Transmission Rates

*Culicoides sonorensis* females infected with the Florida EHDV-2 strain had higher rates of infection than those infected with the Can-Alberta strain. Over the course of the experiment, 145 out of 167 (86.8%) bodies tested positive for the Can-Alberta strain of EHDV-2 and 237 out of 250 (94.8%) bodies tested positive for the Florida strain of the virus. By day three, when sampling began, 65.0% and 94.4% of bodies sampled were positive from Can-Alberta and Florida strains, respectively. Infection rates varied by day (Table 1), reaching 100.0% of individuals for both strains on multiple sampling days. Contingency table analyses indicated significance in infection rates by strain (*χ*^2^ = 7.27, df = 1, *p* = 0.007), time (*χ*^2^ = 20.13, df = 11, *p* = 0.044) and time by strain interactions (*χ*^2^ = 48.80, df = 30, *p* = 0.016). The bodies of control midges (*n* = 15) were also individually tested and all were negative for both strains of the virus.

Overall transmission rates (saliva samples positive for viral RNA from the honey card and capillary tube assays combined) for the Florida strain (92/157, 58.6%) were slightly higher than those for the Can-Alberta strain (47/85, 55.3%). However, overall transmission potential was not significantly different between virus strains (*χ*^2^ = 0.13, df = 1, *p* = 0.719), but varied significantly with time (*χ*^2^ = 51.57, df = 9, *p* < 0.001) and time by strain interactions (*χ*^2^ = 64.93, df = 24, *p* < 0.001). The time taken for 50.0% of infected individuals to have demonstrable virus levels in their saliva (EIP_50_) post-feeding was slightly longer (eight days) for the Florida strain infected midges than for Can-Alberta strain infected midges (seven days). Both strains reached an EIP_75_ on day 10 post-feeding, with high rates of transmission potential (>70.0%) observed through the end of the experiment for both strains with the exception of day 13 post-feeding for the Can-Alberta strain (50.0%).

Rates of disseminated infection of the two strains followed a similar pattern as susceptibility to infection, with higher dissemination rates observed in midges infected with the Florida strain than those infected with the Can-Alberta strain. Dissemination to leg tissues was seen in 110 out of 145 individuals (75.9%) with the Can-Alberta strain and 208 out of 237 individuals (87.8%) with the Florida strain of EHDV-2. Dissemination for both the Can-Alberta strain and Florida strain was recorded as early as day 3 post-feeding, with 23.1% and 29.4% dissemination rates observed in the Can-Alberta and Florida strains, respectively. Also, by day 5 post-feeding, infection and dissemination rates were similar, suggesting a limited midgut escape barrier for these EHDV strains in *C. sonorensis*. Contingency table analyses indicated that dissemination rates were significantly different by virus strain (*χ*^2^ = 8.30, df = 1, *p* = 0.004), time (*χ*^2^ = 93.66, df = 11, *p* < 0.001), and time by strain interaction (*χ*^2^ = 105.15, df = 30, *p* < 0.001). Higher dissemination rates were found for the Florida strain overall, with dissemination seen in more than 50% of individuals on day 4 for Florida strain and day 5 for Canada strain.

### 3.2. Viral Titer Analysis

**Figure 1:**
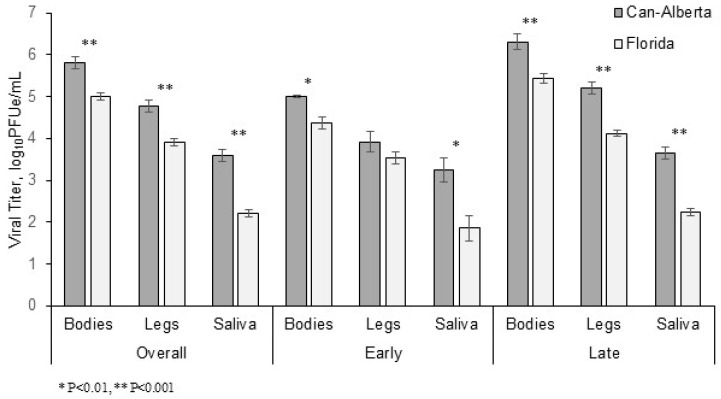
Viral titers in the bodies, legs, and saliva of *Culicoides sonorensis* infected with Can-Alberta or Florida strain of EHDV-2. Overall viral titers, viral titers during the first 3–7 days post-feeding (early), and viral titers during the last 8–13 days post-feeding (late) are shown for both strains. Error bars indicate the standard error of the mean.

Viral titers (log_10_ PFUe/mL) were significantly higher for Can-Alberta strain bodies (t = 4.72, df = 254, *p* < 0.001), legs (t = 5.36, df = 178, *p* < 0.001), and saliva (t = 8.60, df = 79, *p* < 0.001) overall, compared to the Florida strain infected samples (Figure 1). When time periods were split into early infections and late infections, Can-Alberta strain body and saliva titers were both significantly greater than Florida strain titers in midges collected from days 3–8 (body: t = 2.71, df = 109, *p* = 0.008; and saliva: t = 3.38, df = 12, *p* = 0.005), and body, leg, and saliva titers were significantly higher after day 8 (body: t = 4.06, df = 144, *p* < 0.001; leg: t = 6.42, df = 111, *p* < 0.001; saliva: t = 8.03, df = 64, *p* < 0.001). The only non-significant comparison was that of legs during the early time period (t = 1.32, df = 68, *p* = 0.193).

**Figure 2:**
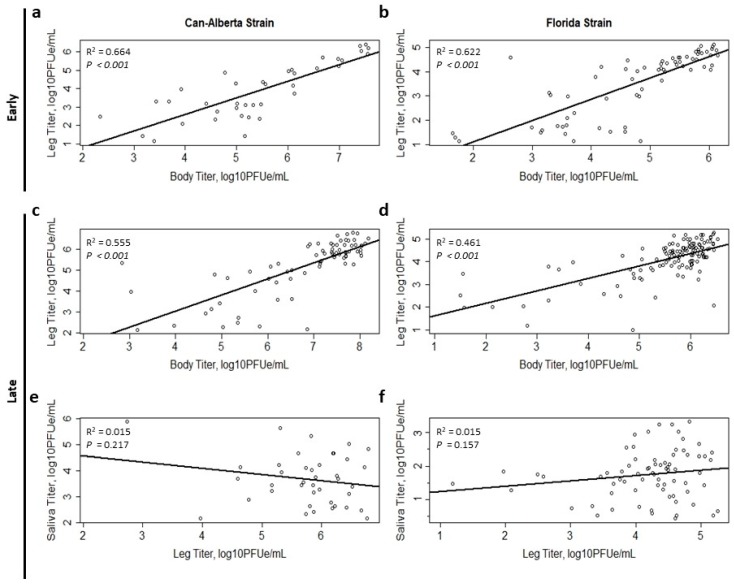
Viral titer relationships between bodies, legs, and saliva of *C. sonorensis* infected with two strains of EHDV-2. (**a**) Body by leg titer correlation for Can-Alberta, (**b**) body by leg titer correlation for Florida strain, (**c**) body by leg titer correlation for Can-Alberta, (**d**) body by leg titer correlation for Florida, (**e**) leg by saliva correlation for Can-Alberta, (**f**) leg by saliva correlation for Florida. Figure 2a,b show correlations during the early period (days 3–7 post-feeding) and Figure 2c–f show correlations during the late period (days 8–13 post-feeding).

Viral titers in the bodies and legs of both the Can-Alberta and Florida strains were significantly positively associated during the early period (Can-Alberta R^2^ = 0.66, *p* < 0.001, Figure 2a; Florida R^2^ = 0.62, *p* < 0.001, Figure 2b) and late period (Can-Alberta R^2^ = 0.55, *p* < 0.001, Figure 2c; Florida R^2^ = 0.46, *p* < 0.001, Figure 2d). The low number of positive saliva samples (N = 13 Can-Alberta and 17 Florida) prohibited the analysis of leg by saliva regression analyses during the early period. Neither Can-Alberta (R^2^ = 0.02, *p* = 0.217, Figure 2e) nor Florida (R^2^ = 0.02, *p* = 0.157, Figure 2f) strains showed a significant correlation between leg and saliva titers during the late period.

### 3.3. Honey Card Versus Capillary Tube Assay Comparison

**Figure 3:**
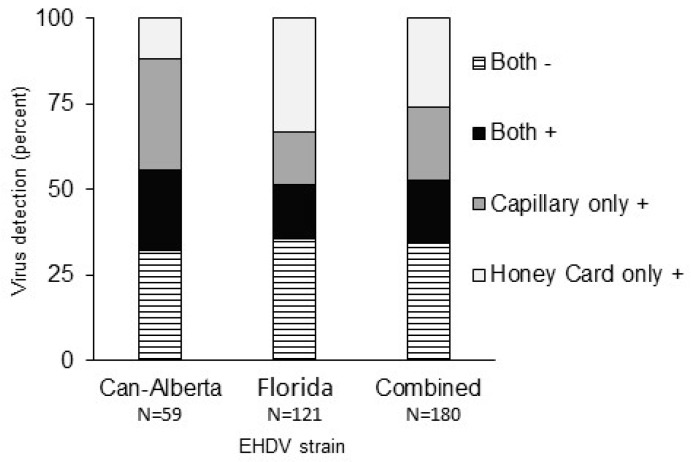
Virus detection by saliva collection method for two strains of EHDV-2 in *Culicoides sonorensis* as a percentage of total salivary assays completed. Consensus between the results of capillary assays and honey cards was reached for 52.8% of samples. Chi-square results indicated that positive detections were significantly dependent upon method, although this result was confounded by strain. Greater detections were made by capillary assays for the Can-Alberta strain and by honey cards for Florida strain infected midges.

For the 59 Can-Alberta strain and 121 Florida strain individuals using data from both assay methods, a significant interaction of virus strain and method was identified (*χ*^2^ = 13.46, df = 3, *p* = 0.004). When strains were analyzed separately, positive results (viral RNA detections) were significantly dependent on the method used for both Florida (*χ*^2^ = 6.88, df = 1, *p* = 0.009) and Can-Alberta strains (*χ*^2^ = 20.12, df = 1, *p* < 0.001). These results were confounded between strains with more positives detected by capillary assays in Can-Alberta strain infected midges (33/59 positive by capillary assay, 21/59 positive by honey card assay) and more positives detected by honey card assay in Florida strain infected midges (38/121 positives by capillary assay, 59/121 positive by honey card assay). Overall, consensus in results between the honey card and capillary tube assays was seen in just over half (52.8%, 95/180) of samples, with 33 individuals testing positive through both methods and 62 individuals testing negative through both methods. Of the remaining 47.22%, capillary assays detected positives in 38 samples and honey cards detected positives in 47 samples (Figure 3). This indicates that both assays resulted in false negatives that could have led to miscalculations of transmission potential between strains had multiple assay methods not been used.

**Figure 4:**
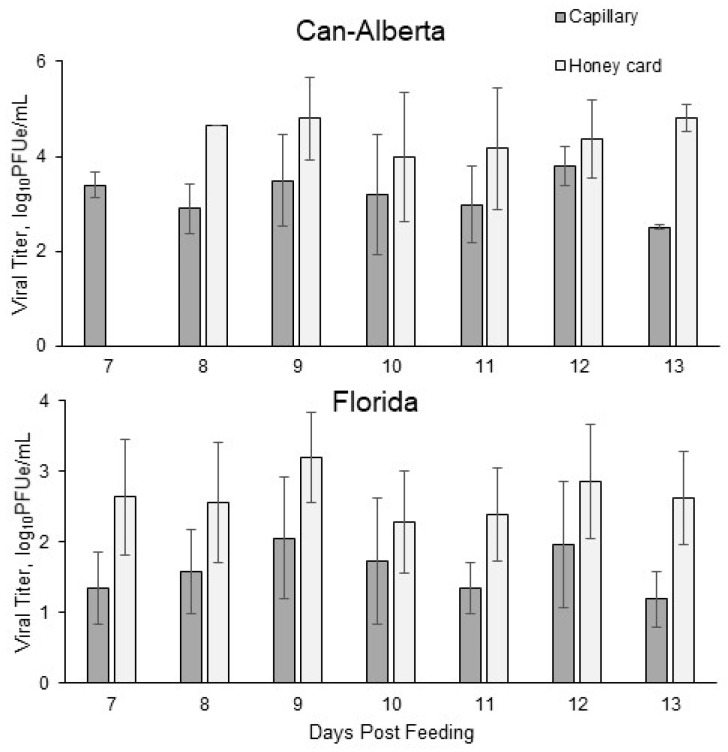
Mean viral titers of Can-Alberta and Florida strains of EHDV-2 in the saliva of *C. sonorensis* collected using honey card or capillary tube assays. Saliva samples were collected daily from days 7–13 post-feeding. *Culicoides sonorensis* individuals were initially fed 5.5 log_10_PFUe/mL of each strain through a blood meal. Error bars show the standard deviation.

Viral titers were also significantly different between the two transmission assay methods examined. Mean viral titer (±σ) of the Florida strain was significantly higher using the honey card method (2.6 ± 0.7 log_10_PFUe/mL) compared to capillary tube assays (1.6 ± 0.7 log_10_PFUe/mL) (t = −6.47, df = 81, *p* < 0.001). Similarly, mean viral titer of the Can-Alberta strain was significantly higher using the honey card method (4.3 ± 1.0 log_10_PFUe/mL) compared to the capillary tube method (3.2 ± 0.8 log_10_PFUe/mL) (t = −4.74, df = 38, *p* < 0.001). This trend was observed in saliva collections throughout the saliva collection period (7–13 days post-feeding) except on day 7 when no Can-Alberta honey cards were positive (Figure 4).

## 4. Discussion

Although differences in the vector competence of *Culicoides* species to multiple serotypes has been documented [17,43,44], our data indicate that key aspects of vector competence are also significantly affected by different viral strains within the same serotype. Infection and dissemination rates of the Florida strain of EHDV-2 were significantly higher in *C. sonorensis* than the reference Can-Alberta strain. Despite this difference, the Can-Alberta strain produced an overall higher titer of virus in *C. sonorensis* at all stages of infection, including in the body, legs, and saliva. It is currently unclear why an overall higher replicative advantage in viral titer in the Can-Alberta strain did not produce similar or higher infection, dissemination, and transmission rates than the lower propagation of viral titer in the Florida strains of EHDV-2. Nonetheless, these results suggest that arbovirus replication kinetics are only partially responsible for negotiation of infection barriers in *Culicoides.* It is possible that strength of the midgut infection barrier, midgut escape barrier, and/or dissemination barrier of *C. sonorensis* varies with respect to the viral strain, which in this case *C. sonorensis* may exhibit comparatively stronger barriers to infection towards the Can-Alberta strain than towards the Florida strain [45]. However, further studies will be needed to test this hypothesis and to also examine if/how the immune response of the midge varies when orally challenged with different strains and serotypes of an Orbivirus. Previous studies comparing two strains of West Nile virus (WNV) infection in mosquitoes observed similar patterns between rates of susceptibility to infection and association with viral titer [46]. Specifically, WNV strain FIN infected *Aedes japonicus* Theobald at significantly lower rates than WNV strain NY99, despite the former strain producing higher titer infections. Along the same lines, high viral titers of WNV strains in bodies and legs were not associated with high transmission rates in *Culex quinquefasciatus* Say [47]. Taken together, these observations suggest that different virus strains have different affinities to specific cells and organs within the arthropod and that viral titer is not always an accurate predictor of viral infection progression in the vector.

Although *C. sonorensis*, in a broad sense, is a confirmed vector of EHDV, the extremely high infection and dissemination rates seen for both strains of EHDV-2 were unexpected. The infection rates seen in other studies investigating the same colony strain of *C. sonorensis* at 25 °C for EHDV-2 have been variable. Two other studies found average daily infection rates of 52.3% and 70.3% (from day three through the end of each experiment) with infectious blood meal titers of 6.9 log_10_PFUe/mL and 6.8 log_10_TCID_50_, respectively [42,48]. This is compared to the present study where the average daily infection rate from day three onward was 88.9% for Can-Alberta and 95.2% for Florida strain infected midges fed 5.5 log_10_PFUe/mL. It is possible that this variation in the infection rates observed is due to differences in the virus strains used between the studies. Previous studies used EHDV-2 strains isolated from West Virginia and Kansas whereas we used the Can-Alberta and Florida Strains in this study. Nonetheless, these findings collectively suggest that the outcome of an infectious blood meal varies with the EHDV strain used and highlight the importance of considering the origin of viral strain when designing vector competence experiments and interpreting/extrapolating the results across a broad spectrum of geographically distinct viral strains.

The high infection, dissemination, and transmission rates observed in the present study also have implications for understanding barriers to transmission of EHDV within this species. *Culicoides sonorensis*, which is also a documented bluetongue virus vector [49], was shown to have a midgut infection barrier, midgut escape barrier, and a dissemination barrier, but lack of a salivary gland infection or salivary gland escape barrier to bluetongue virus [50]. The overall high infection and dissemination rates seen at 3 days post-feeding and thereafter indicate weak midgut infection, midgut escape, and dissemination barriers in *C. sonorensis* to both strains of EHDV used in this study. Transmission rates were moderately high, with 55.3% of Can-Alberta infected midges and 58.6% of Florida infected midges having disseminated infections producing EHDV-positive saliva. Although these overall transmission rates are considerably lower than the overall infection and disseminated infection rates, transmission rates increased considerably for both strains at ten days post-feeding, indicating that a salivary gland barrier is unlikely for either strain of EHDV-2 in *C. sonorensis.*

Greater transmission potential identified in midges that survived one week post blood feeding (pbf) has a significant effect on the calculation of vectorial capacity of *C. sonorensis* for both strains. These “one-week” pbf insects are more likely to have completed the extrinsic incubation period and have virus present in the salivary glands and are therefore more likely to transmit the pathogen than younger insects earlier in the infection process [30,51]. We identified an EIP_50_ of 7–8 days and an EIP_75_ of 10 days post infection with high levels of infection sustained throughout the completion of the experiment. While temperature was known to affect EIP in *C. sonorensis*, with higher temperatures leading to shorter EIP length [48], we found that viral strain can also affect EIP and will impact calculations of vectorial capacity for *C. sonorensis* with EHDV. It is important to note that no Can-Alberta infected *C. sonorensis* survived to day 14 pbf. This result is likely the combination of lower initial blood-feeding rates for this strain as well as natural mortality throughout the experiment. However, this result may also be attributable to potentially deleterious effects of higher titer infections experienced by the Can-Alberta infected midges. If high titer infections resulted in the observed mortality, this may significantly alter the vectorial capacity of *C. sonorensis* populations infected with this viral strain by decreasing their daily probability of survival. Additional studies should be employed to better understand the negative effects of high titer infections of EHDV-2 on infected *Culicoides* and the resulting impact on calculations of their vectorial capacity.

Although rates of infection and dissemination were overall higher for the Florida strain infected midges, higher viral titers were recovered from individuals infected with the Can-Alberta strain of the virus. This finding is an important consideration for the overall transmission potential between the two strains. The average viral titer of EHDV-2 in the saliva of Florida strain-infected *Culicoides* was 2.2 log_10_PFUe/mL (3.2 log_10_TCID_50_) and that of Can-Alberta infected midges was much higher, at 3.6 log_10_PFUe/mL (5.1 log_10_TCID_50_), both of which were above the accepted transmission threshold of 2.7 log_10_TCID_50_ [43,52]. One study investigating the transmission of bluetongue virus to sheep by *C. sonorensis* found that greater viral titers found in infected midges resulted in greater antibody responses and higher titer viremia in sheep [53]. While both viral strains surpass the viral threshold for transmission, the overall higher titer of virus present in the saliva of Can-Alberta infected midges may make this strain more virulent to susceptible vertebrate hosts. However, further studies will be needed to test this hypothesis. Moreover, *C. sonorensis* is not found in great abundance in the state of Florida [54,55,56,57,58], suggesting that the infection, dissemination, and transmission dynamics of the Florida strain may depend on potential coevolution of this strain with other locally abundant vectors. Investigating the transmission rate and virus titer of the Florida strain in the more abundant local *Culicoides* species that the virus may have coevolved with should provide greater insight into the transmission dynamics of the EHDV-2 strain in Florida.

Honey FTA cards have been successfully employed for the detection of virus transmission in mosquitoes and *Culicoides* species [29,30,31]; however, nucleic acid preserving agents present in FTA cards have been linked to mortality in mosquitoes, indicating that this substrate may not be suitable for all virus transmission studies [30]. The present study showed that EHDV can be collected and detected using plain Whatman filter papers without the need for specialized preserving agents, which has significant implications in future vector competence studies. Capillary assays rely on the survival of the vector until harvesting as this is an end-stage assay that can only be performed on a living individual immediately preceding death, with capillary assays relying on destructive sampling of the insect [59,60]. Comparatively, collection of saliva using honey cards is a non-destructive sampling methodology, which may be utilized in a repeated analysis format in the long term. More importantly, the honey card approach is not a terminal assay and can be employed throughout an infection study to monitor for first transmission events without the need to harvest the individual [37,61,62]. This non-destructive sampling technique enables investigators to test the same individual midge multiple times and to calculate the extrinsic incubation period on a per capita basis. In addition, due to challenges associated with maintaining field-collected *Culicoides* adults in laboratory environments for extended periods of time along with the considerable skill level required to perform these techniques, the capillary tube bioassay may be limited by sample sizes. In contrast, the honey card approach is much less labor-intensive and allows for high throughput analyses of the saliva collected from midges. Moreover, use of this method could result in comparatively greater sample sizes in future vector competence studies in *Culicoides* species when potentially working with field-collected individuals. Regardless, the honey card method was found to be only significantly better for Florida infected midges in the present study. It is unclear why confounding results were found in the transmission methods between strains; however, for both strains, a large proportion of transmission events were detected only by a single method. Based on the results of our methods comparison and the inability of either method to detect every positive result, using multiple methods to detect transmission events may provide a more accurate representation of the arbovirus transmission rate in insect vectors [37,38].

## 5. Conclusions

The present study demonstrated the infection susceptibility of *Culicoides sonorensis* for an actively circulating strain of EHDV-2 isolated from the Florida panhandle region. When compared with a reference strain of EHDV-2 from Alberta, Canada, rates of infection and dissemination were significantly higher for the Florida EHDV strain, while viral titers were higher for the Can-Alberta strain. These results suggest that strain of the virus can significantly influence vector competence parameters of *Culicoides* species and, therefore, should be considered a fundamental aspect of vector competence studies involving *Culicoides* species and Orbiviruses.

## Figures and Tables

**Table 1:** Daily infection, dissemination, and transmission percentages (capillary tube and honey card methods combined) for the Can-Alberta strain and the Florida strain of EHDV-2 in *Culicoides sonorensis*. The number that tested positive over the total number assayed is included in parenthesis.

	Can-Alberta Strain			Florida Strain		
Day	Infection ^a^	Dissemination ^a^	Transmission ^a^	Infection ^a^	Dissemination ^a^	Transmission ^a^
3	65.0 (13/20)	23.1 (3/13)	nd	94.4 (17/18)	29.4 (5/17)	nd
4	80.0 (12/15)	41.7 (5/12)	nd	94.4 (17/18)	76.5 (13/17)	nd
5	91.7 (11/12)	90.9 (10/11)	22.2 (2/9)	91.1 (22/24)	86.4 (19/22)	25.0 (1/4)
6	91.7 (11/12)	100.0 (11/11)	18.2 (2/11)	94.1 (16/17)	87.5 (14/16)	0.0 (0/14)
7	100.0 (14/14)	85.7 (12/14)	58.3 (7/12)	100.0 (18/18)	94.4 (17/18)	35.3 (6/17)
8	76.9 (20/26)	70.0 (14/20)	50.0 (5/10)	100.0 (19/19)	100.0 (19/19)	52.6 (10/19)
9	100.0 (19/19)	84.2 (16/19)	66.7 (8/12)	100.0 (18/18)	88.9 (16/18)	53.3 (8/15)
10	84.2 (16/19)	87.5 (14/16)	75.0 (6/8)	91.3 (21/23)	90.5 (19/21)	79.0 (15/19)
11	100.0 (10/10)	90.0 (9/10)	87.5 (7/8)	87.5 (21/24)	95.2 (20/21)	75.0 (12/16)
12	100.0 (11/11)	81.8 (9/11)	87.5 (7/8)	88.9 (24/27)	100.0 (24/24)	70.6 (12/17)
13	88.9 (8/9)	87.5 (7/8)	50.0 (3/6)	100.0 (23/23)	91.3 (21/23)	75.0 (15/20)
14 ^b^	nd	nd	nd	100.0 (21/21)	100.0 (21/21)	81.3 (13/16)

Nd = not determined; ^a^ infection, dissemination, and transmission potential rates are reported in percentages; ^b^ no Can-Alberta infected individuals survived to day 14 post-feeding.

## Supplementary Materials

Supplementary materials can be found at https://www.mdpi.com/1999-4915/11/4/367/s1.
