# Supplementary material for: Vector Competence of Culicoides sonorensis (Diptera: Ceratopogonidae) for Epizootic Hemorrhagic Disease Virus Serotype 2 Strains from Canada and Florida
Source: Viruses. 2019 Apr 22;11(4):367. doi: 10.3390/v11040367 (PMC6521025; doi:10.3390/v11040367)

Supplementary File 1: Standard viral curves for the Can-Alberta strain (a) and Florida strain (b) of EHDV-2 used to experimentally infect colony *Culicoides sonorensis*. Viral curves were generated using serially diluted virus samples for each strain. Plaque assays were run to determine PFU/mL and compared to Cq values generated using qRT-PCR. Both viral curves were generated using Bio-Rad CFX Manager 3.1 software.

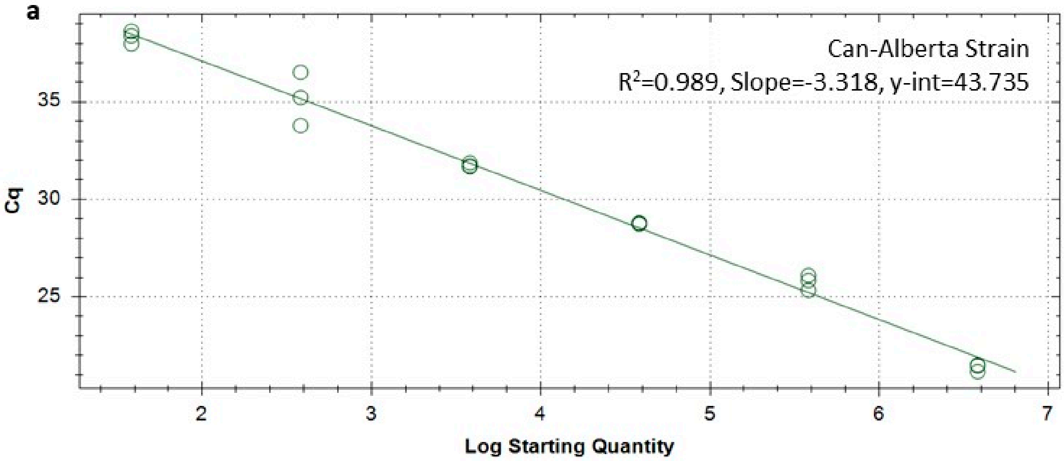

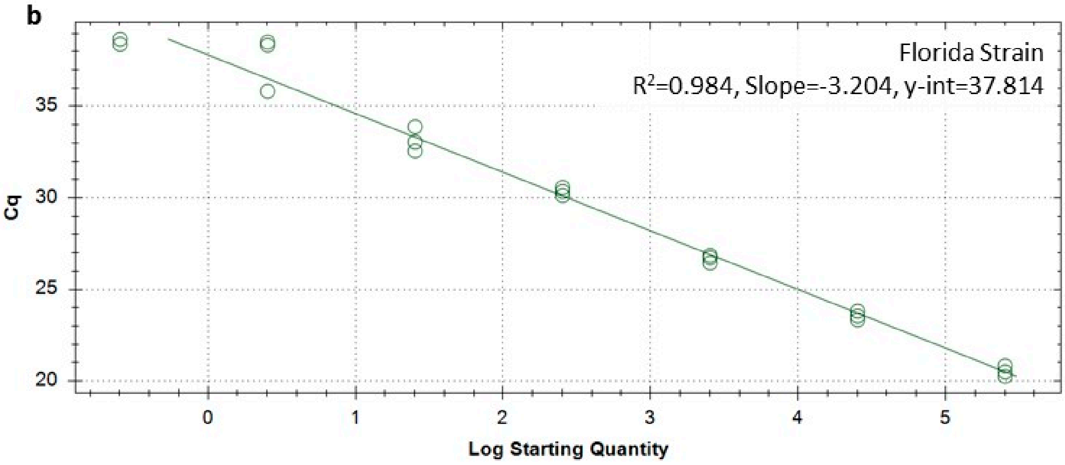

Supplement: Supplementary file 1 [file viruses-11-00367-s001.pdf]
